# Dynamic changes of intestinal flora in patients with irritable bowel syndrome combined with anxiety and depression after oral administration of enterobacteria capsules

**DOI:** 10.1080/21655979.2021.1999374

**Published:** 2021-12-25

**Authors:** Qingqing Guo, Hao Lin, Pengcheng Chen, Songlin Tan, Zhiyong Wen, Lijian Lin, Jianquan He, Jianbo Wen, Shiyun Lu

**Affiliations:** a Department of Intensive Medicine, The First Affiliated Hospital of Fujian Medical University, Fuzhou, Fujian, China; b Shengli Clinical Medical College, Fujian Medical University, Fuzhou, Fujian, China; c Department of Gastroenterology, Fujian Provincial Hospital South Branch, Fuzhou, Fujian, China; d The First School of Clinical Medicine, Southern Medical University, Guangzhou, Guangdong, China; e Department of Health Management, Fujian Provincial Hospital South Branch, Fuzhou, Fujian, China; f Department of Gastroenterology, Affiliated Ping Xiang Hospital, Southern Medical University, Pingxiang, Jiangxi, China; g Department of Emergency, Fujian Provincial Hospital, Fujian Medical University, Fuzhou, Fujian, China; h School of Medicine, Xiamen University, Xiamen, China

**Keywords:** Diarrheal irritable bowel syndrome, fecal microbiota transplantation, anxiety and depression, bacterial community

## Abstract

This study investigated the clinical characteristics and dynamic changes of intestinal bacterial community to evaluate the curative effect of fecal microbiota transplantation (FMT) on irritable bowel syndrome with predominant diarrhea (IBS-D) comorbid with anxiety and depression. Total two treatments were designed in randomize-controlled trial includes oral FMT capsules with 1 week (A1), 8 weeks (A2), and 12 weeks (A3), as well as oral empty capsules with 1 week (B1), 8 weeks (B2), and 12 weeks (B3) as control for comparison. The positive therapeutic effects occurred in FMT colonized patient with IBS-D comorbid psychological disorder, demonstrated at alleviated IBS-D severity (IBS-SSS score from 291.11 reduced to 144.44), altered stool type (from 6 changed to 4), reduced anxiety and depression scores (from 18.33 to 8.39 and from 22.33 to 17.78) after FMT-treated 12 weeks. The FMT therapy improved bacterial alpha diversity and the majority bacterial community predominant by *Bacteroidetes* and *Firmicutes*, and the relative abundance (RA) was higher after FMT-treated 12 weeks (50.61% and 45.52%) than control (47.62% and 38.96%). In short, FMT therapy has great potential for IBS-D patients combined with anxiety and depression by alleviated clinical symptoms and restore the intestinal micro-ecology.

## Graphical abstract

**Figure:**
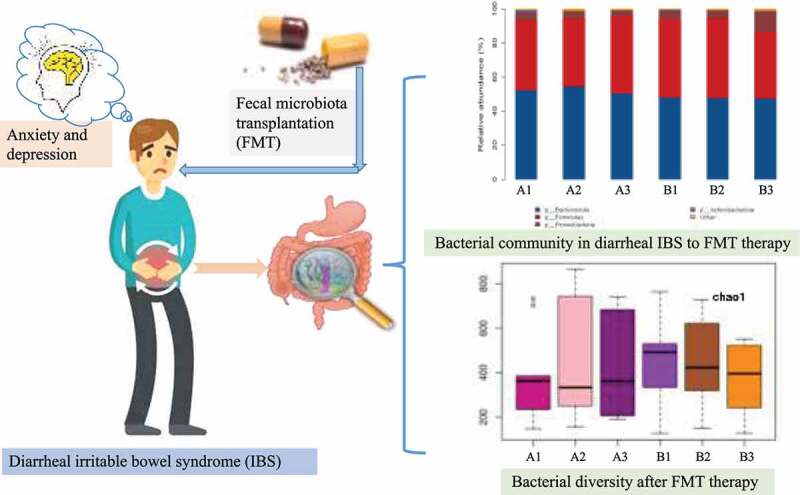

## 1. Introduction

Irritable bowel syndrome (IBS) is a persistent or intermittent functional-gastrointestinal disorder and characterized by altered bowel habits as well as abdominal pain. According to statistics, the global IBS prevalence rate ranged from 7% to 21%, and approximately one third of patients are dominant by constipation, and one third suffer from diarrhea, and the rest patients are mixed or unclassified bowel pattern [1,2]. Previous study has recognized the gastrointestinal diseases usually overlap with other dysfunctions and psychiatric diseases, as estimated that 60–85% of clinical gastrointestinal patients suffer from mental disorders. There seems to be bidirectional between IBS and psychiatric diseases, and it is reported that the prevalence of depression in IBS patients is as high as 84% and the prevalence of anxiety is 44%; meanwhile, 25–30% of depression and 10–45% of anxiety patients will be developed into IBS [3]. Since the depression contributed as the major factor of disability worldwide, anxiety is also ranked the sixth largest actor to the non-fatal burden. Therefore, the co-occurrence of chronic diseases and psychiatric disorders is a huge burden on patients.

The pathogenesis of IBS is still unclear and the destruction of symbiosis between human hosts and microbial communities, and the comprehensive interaction or communication between microbial communities and autonomic nervous system is considered to be the core factor for the persistence of IBS symptoms [4,5]. Importantly, the intestinal microbiota is known as second brain of humans and essential in regulating the central nervous system [6,7]. Recent studies have hypothesized that the ecological disorders and immune responses observed in IBS may drive and maintain the IBS gastrointestinal symptoms [4]. As reported, the fecal microbial characteristics are similar between irritable bowel syndrome with predominant diarrhea (IBS-D) and depression patients, while lower microbial diversity observed compare with healthy donors and speculated to be the mucosal immune barrier dysfunction derived by microbial disorders [6–8]. Pittayanon et al. found higher abundance of *Proteobacteria* and *Bacteroides* in IBS with psychiatric disorders patients, while lower alpha diversity and abundance of *Bacteroidetes, Actinobacteria*, and *Faecalibacterium* [9,10]. Therefore, to balance the intestinal microbial ecosystem, researchers keep positively and constantly exploring pharmacological and non-pharmacological treatments comprised diet change, antispasmodics, anti-diarrhoeals, antidepressants, probiotics, antibiotics, serotonin agonists, hypnotherapy, and guanylate cyclase C agonists intervention [11–13].

Among them, the technique of fecal microbiota transplantation (FMT) has been proposed through transferring the healthy donors gut microbiota to recipient, aim to restore the imbalanced microbial communities, and establish a complex and stable microbial community system [14,15]. In current clinical practice, FMT has been proved to be very successful to treat *Clostridium difficile* infection with 90% cure rate [16,17]. However, the efficiency of FMT to treat IBS is still controversial, Kurokawa et al. suggested that FMT may effectively alleviate certain mental disorders after 8 weeks oral microbial transplantation in autistic children [3]. While Aroniadis et al. pointed out that hadn’t relief symptoms after 12 weeks FMT treated on IBS-D through a randomized, placebo-controlled and double-blind trial [18]. However, a cohort trial observed the improvement in IBS-SSS-based symptoms after 3 months of FMT treatment [19–22].

Although previous study confirmed that oral FMT have been used for IBS therapy, while there is no specific treatment for IBS-D comorbid with anxiety and depression, as well as limited information in intestinal bacterial community dynamics response to FMT treatment. Therefore, the present study investigates clinical symptoms and detected the bacterial community dynamics through 16S rRNA Miseq_PE300 sequencing and bioinformatics analysis on fecal samples of FMT-treated IBS-D patients compared with control, aimed at evaluating the curative effect of FMT on alleviated clinical symptoms and reconstruction of intestinal micro-ecology in IBS-D and psychiatric comorbidities patients.

## 2. Materials and methods

### 2.1. Participant’s recruitment

Present study was carried out at Ping Xiang People’s Hospital in Jiangxi, China. Patients diagnosed of IBS-D as well as Hamilton anxiety and depression score within 14–28 and 20–34 points were recruited for this trial. The exclusion criteria were as follows: had any abdominal surgery, human immunodeficiency virus infection, kidney disease, psychosis (mania and schizophrenia), pregnancy, active infection, abnormal thyroid function, abnormal liver function, use of probiotics, prebiotics, and antibiotics in the last 2 weeks, as well as participate in other clinical trials within 3 months were excluded. Total 18 patients were included to evaluate the response of fecal bacteria transplantation to clinical symptoms and dynamic changes of intestinal bacteria after FMT treatment. This study protocol was registered in China Clinical Trial Registry Center (ChiCTR1900024924) and approved by the Ethics Committee of Pingxiang People’s Hospital (2019R001-F04). The informed consent and voluntarily accept fecal bacteria transplantation of participants was obtained before enrollment.

### 2.2. Trial design as well as feces collection

Present study belongs to randomized-controlled observational clinical trial, and 18 patients diagnosed as IBS-D comorbid with anxiety and depression were randomly divided into two group of FMT therapy and control (9 patients in each group). The FMT treatment was intervened by oral enteric capsules for 3 times (every 2 days one time and 30 capsules each time) and recorded the symptoms after therapeutic 1 week (A1), 8 weeks (A2), and 12 weeks (A3). At the same time, oral empty capsules as control and record the symptoms after 1 week (B1), 8 weeks (B2), and 12 weeks (B3). Enterobacter FMT capsules and empty capsules were technical supported by Chengge Biotechnology Co., Ltd. (Xiamen, China). The feces collection was carried out by using the feces collection box provided from Allwegene Technology Co., Ltd (Beijing, China).

### 2.3. 16S rRNA Miseq_PE300 sequencing

After completing the genomic DNA extraction from collected feces samples, quality was determined by using 1% agarose gel electrophoresis. The specific primers with barcode were synthesized according to the target sequencing region V3-4, and the sizes of the amplified bands of polymerase chain reaction products were detected by 1% agarose gel electrophoresis and purified by Agencourt AMPure XP nucleic acid purification kit. Furthermore, Miseq library construction and computer sequencing was by Allwegene Technology Co., Ltd (Beijing, China).

### 2.4. Bioinformatics and statistical analysis

The obtained sequencing from Miseq sequencing was Pair-End (PE) double-ended sequence data; the measured Fastq data perform quality control by using Trimmomatic (v0.36) and Pear (v0.9.6), and then merged the two ends of the sequence according to the overlap relationship of PE by using Flash (v1.20) and Pear (v0.9.6), remove the chimera of the Fasta sequence and short sequence, finally obtained high quality Fasta data. The valid data from Raw PE remove barcode and primer obtained raw tags further remove chimera and short sequences get clean tags. Clustering all clean tags and classified Operational Taxonomic Units (OTUs) according to different similarity levels, then perform biological information statistical analysis by Qiime (Version 1.8.0 http://www.qiime.org/) and Vsearch (2.7.1) at 97% similarity level.

Bacterial annotation taxonomy analysis obtained from blast or RDP Classifier compare the representative sequences of OTUs and the annotated at phylum, class, order, family, and genus levels. The heatmap was obtained from distance calculation and clustering analysis by R language (vegan packages), vegdist, and hclust. Alpha diversity comprised of Chao1, Simpson, Observed species, PD whole tree, and Shannon were calculated by qiime (v.18.0). Beta diversity based on unifrac and Bray–Curtis of principal co-ordinates and component analysis as well as non-metric multidimensional scaling carried out by R language. The network analysis was performed during top 20 genera by using spearman method in R language.

For clinical indicators, the severity of abdominal symptoms was evaluated by irritable bowel syndrome scoring system (IBS-SSS) and classified as mild, moderate, and severe (range of 75–175, 175–300, 300–500) [22,23]. The psychiatric symptoms of patients were evaluated by trained psychiatrists using Hamilton Anxiety and Depression Rating Scale (HAM-A and HAM-D). Based on American Psychiatric Association guidelines, the normal range of anxiety and depression were HAM-A < 14 and HAM-D < 8 [24]. Additionally, Bristol Stool Form Scale was used as feces shape classification (7 = liquid, 3 and 4 represent ideal shape, 1 = very hard), and quality of life was assessed by irritable bowel syndrome quality of life Scale (IBS-QOL) [25–27].

## 3. Results and discussion

### 3.1. Clinical response of IBS-D patients to fecal microbiota transplantation therapy

To evaluate the curative effect of FMT on clinical symptoms relief of IBS-D severity and anxiety/depression, the distribution of clinically characters is demonstrated in Tables 1 and 2. A total of 18 patients were enrolled and compared 9 FMT treated and 9 without FMT-treated IBS-D patients in randomized controlled trials. The stool samples were collected from all of the patients in the FMT group or placebo group. Moreover, the FMT group consisted of 5 males and 4 females with a mean age of 44 years, while 5 males and 4 females with an average age of 50 years in the control group. The positive clinical response occurred in FMT group, while the clinical symptom was similarly between two groups at the initially stage. Specifically, the mean score of IBS-SSS and stool type were 284.44 and 6 in control, 291.11 and 6 in FMT treatment, anxiety and depression mean scores were 19.28 and 22.17 in control, 18.33 and 22.33 for FMT treatment, and quality of life score was 44.11 and 43.33 separately. After 1 week of follow-up, the curative effect of FMT was obviously appeared and manifested as declined scores of IBS-SSS (215.56), HAM-D (19.94), and HAM-A (13.5), and quality of life score was 40, and at the same time, the stool characteristics was improved (Table 1). After follow-up of 4 weeks, obviously clinical improvement comprised of decreased IBS-SSS score (166.67) and stool type (5), anxiety and depression score (9.11 and 18.61), while quality of life was 27.56, which indicated that FMT takes a certain period of time to improve intestinal motility and relieve the IBS-D symptoms. Thereafter, clinical symptom kept steady with IBS-SSS score of 144.44–177.78, anxiety and depression score of 8.39–9 and 17.78–18.5, stool type classified 4–5, and quality of life score was 24.67–23 after FMT-treated 8–12 weeks (Table 2).

**Table 1.** The distribution of clinically characters during 0–1 week in irritable bowel syndrome with predominant diarrhea (IBS-D) patients after fecal microbiota transplantation therapy

No.	Age	Gender	Treatment	Anxiety	Depression	IBS-SSS	Stool classification	Quality of life	Anxiety	Depression	IBS-SSS	Stool classification	Quality of life
				Week 0					Week 1				
1	69	Male	Control	20.5	20	280	6	53	20	22	260	6	52
7	59	Female	Control	20.5	21.5	220	6	40	15	22	220	6	40
10	65	Male	Control	17	20.5	320	6	40	14.5	21	300	6	40
12	48	Male	Control	14.5	20.5	320	7	42	13.5	19	300	6	41
13	38	Male	Control	23.5	22.5	300	6	56	24	20.5	340	6	55
17	43	Female	Control	28.5	25.5	340	7	50	29	24.5	320	7	50
18	50	Male	Control	14.5	20.5	220	6	32	13.5	19.5	220	6	33
19	42	Female	Control	18	25	260	7	48	17	22.5	240	7	48
21	40	Female	Control	16.5	23.5	300	7	36	16	24.5	280	7	37
2	42	Female	FMT	24.5	24	260	6	51	15	22.5	180	6	49
4	35	Male	FMT	15	21.5	260	7	48	11.5	18.5	200	5	37
5	39	Male	FMT	23	21.5	360	5	48	18	24.5	240	5	45
6	63	Male	FMT	16.5	27.5	260	6	28	13.5	20	220	5	28
9	47	Male	FMT	18.5	22	300	5	44	12	21	200	5	41
14	39	Female	FMT	17.5	22	320	6	40	12.5	18	240	5	40
15	36	Female	FMT	18	20.5	340	7	54	12	16.5	240	5	48
16	58	Male	FMT	15.5	20	220	6	38	14.5	19	180	3	35
20	40	Female	FMT	16.5	22	300	6	39	12.5	19.5	240	4	36

**Table 2.** The distribution of clinically characters during 4–12 week in IBS-D patients after fecal microbiota transplantation therapy

No.	Age	Gender	Treatment	Anxiety	Depression	IBS-SSS	Stool classification	Quality of life	Anxiety	Depression	IBS-SSS	Stool classification	Quality of life	Anxiety	Depression	IBS-SSS	Stool classification	Quality of life
				Week 4					Week 8					Week 12				
1	69	Male	Control	13	23	260	6	48	17	22	200	6	42	12	20	200	6	38
7	59	Female	Control	16.5	24	180	5	36	14	20.5	220	5	34	13	21.5	200	6	36
10	65	Male	Control	13.5	16	300	6	38	13.5	17.5	230	5	39	13.5	22	280	5	29
12	48	Male	Control	12	22	280	6	40	12	23	300	5	38	13	25	300	6	37
13	38	Male	Control	21.5	20.5	320	6	52	20	22.5	300	6	48	18	25.5	300	6	48
17	43	Female	Control	28.5	27.5	320	7	42	28.5	22	300	7	32	28	20	300	7	34
18	50	Male	Control	12	15.5	240	5	28	15	17.5	260	6	30	16.5	18	260	6	30
19	42	Female	Control	16	23	280	6	34	17	24.5	220	7	29	15	28	240	7	34
21	40	Female	Control	15.5	23.5	280	6	38	13	22	280	7	36	16.5	22.5	280	7	35
2	42	Female	FMT	13.5	20	160	6	40	11	19.5	140	5	28	16	18.5	220	6	18
4	35	Male	FMT	2	13.5	180	5	22	5	13.5	160	4	14	4	13.5	160	4	4
5	39	Male	FMT	16	21.5	280	7	32	14	22.5	220	5	28	15.5	25.5	240	5	45
6	63	Male	FMT	10.5	18	160	6	21	10	17.5	140	5	26	8	18.5	160	5	22
9	47	Male	FMT	9.5	20	140	5	28	8	19	120	4	28	9	18	160	4	28
14	39	Female	FMT	9.5	18.5	160	5	28	8.5	18.5	140	5	27	9	18	200	5	28
15	36	Female	FMT	9.5	19	120	4	32	10	17.5	160	5	24	9	18	200	5	24
16	58	Male	FMT	3.5	21	120	3	24	3	16.5	100	3	26	3	18	120	3	18
20	40	Female	FMT	8	16	180	4	21	6	15.5	120	4	21	7.5	18.5	140	4	19

The primary clinical response of FMT-treated IBS-D patients was IBS-SSS score declined by 113 points after 12 weeks, simultaneously the anxiety and depression scores decreased 9 and 4 points. In contrast, the control basically remained stable or slightly increased among all indexes. Schmulson et al. reported the clinical 50 points decrease in IBS-SSS while no difference in IBS-QOL and depression Scale (HADS) after 12 weeks FMT capsule therapy [28], but Xu et al. observed 16% IBS-QOL improvement [29]. Although Kurokawa et al. detected the relief of depression and anxiety after FMT treated while gastrointestinal symptom didn’t positively response [3], but El-Salhy et al. pointed out that abdominal symptoms and quality of life were improved (47.3% and 58.2%) in 60 g FMT treatment and mitigated the IBS severity (IBS-SSS score of 156.4–189.2) [19]. Additionally, Pittayanon et al. reported the significant relief of IBS symptom and improved life quality, while the efficiency declined after some time, therefore suggested that the FMT treatment needs to be repeat provided periodically [10]. From the clinical response of IBS-D patients to FMT treatment, stool characteristics were improved and alleviated the severity of IBS-D combined with anxiety and depression.

### 3.2. Responses of dominant bacterial community in IBS-D to fecal microbiota transplantation therapy

**Figure 1.**
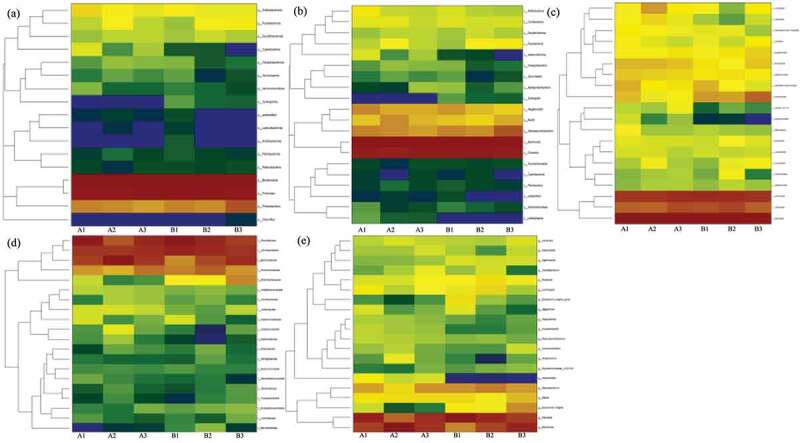
The heat map demonstration of dominant bacterial community response of different fecal microbiota transplantation (FMT) treated duration-based Phylum (a), Class (b), Order (c), Family (d), and Genus (e) level. The FMT capsuletreated irritable bowel syndrome with predominant diarrhea (IBS-D) patients 1 week (A1), 8 weeks (A2), and 12 weeks (A3), as well as control with oral empty capsules 1 week (B1), 8 weeks (B2), and 12 weeks (B3)

This study identified the dominant bacterial community during all samples composed of *Bacteroidetes* and *Firmicutes*, mainly distributed in genus *Bacteroides* and *Prevotella* while distinct with therapy duration (Figure 1). In detail, the *Bacteroidetes* was predominant at the phylum level and the relative abundance (RA) was higher in FMT treatments than control. Notably, the RA of *Bacteroidetes* was increased from 52.43% (A1) to 54.52% (A2), then dropped to 50.61% (A3) with the prolongation of FMT therapeutic duration, while slightly decreased from 48.00 (B1) to 47.62% (B3) in control. The abundance of *Firmicutes* was lower in FMT-treated patients with 1 week (40.99%) and 8 weeks (39.99%) than control (46.12% and 47.09%), while the RA of *Firmicutes* was higher after FMT-treated 12 weeks (45.52% in A3) than control (38.96% in B3). By contrast, lower abundance of *Proteobacteria* (3.02%) and *Actinobacteriota* (0.35%) were identified after FMT treated 12 weeks than control (12.24% and 0.37%). Correspondingly in the class level, the predominant bacterial community was *Bacteroidia* and *Clostridia* during all patients. The proportion of *Bacteroidia* decreased from 52.44% (A1) to 50.61% (A3) in FMT treatments and from 48.00% (B1) to 47.62% (B3) in control, and *Clostridia* ascended from 35.07% (A1) to 41.21% (A3) in FMT treatments while declined from 41.30% (B1) to 35.28% (B3) in control. However, the abundance of *Negativicutes* was decreased in FMT treatments from 4.45% (A1) to 2.36% (A3) and from 3.39% (B1) to 1.23% (B3) in control.

**Figure 2.**
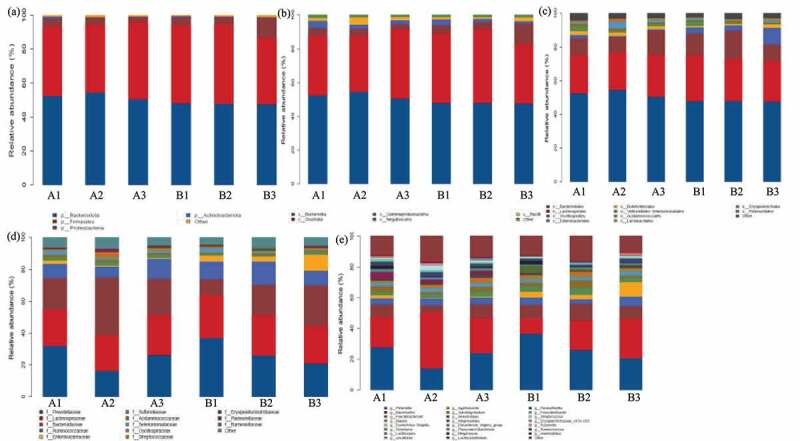
The dominant bacterial community under different fecal microbiota transplantation (FMT) treated duration at Phylum (a), Class (b), Order (c), Family (d), and Genus (e) level. The FMT capsuletreated IBS-D patients 1 week (A1), 8 weeks (A2), and 12 weeks (A3), as well as control with oral empty capsules 1 week (B1), 8 weeks (B2), and 12 weeks (B3)

At the order level, higher abundance of *Bacteroidales* identified in FMT treatments (50.60–54.51%) than control (47.62–47.99%) and *Lachnospirales* was quite similar (22.29–24.68%) between two groups. The higher relative abundance of *Oscillospirales* identified in FMT treatments and increased from 9.91% (A1) to 14.68% (A3) while decreased from 12.85% (B1) to 10.00% (B3) in control; meanwhile, lower abundance of *Enterobacterales* detected in FMT treatments and decreased from 1.97% (A1) to 0.61% (A3) but increased in control from 3.78% (B1) to 10.21% (B3). As to family level, *Prevotellaceae* fluctuated from 31.92% (A1), 16.22% (A2) to 26.41% (A3) in FMT treatments, while continuous declined from 36.78% (B1) to 26.02% (B2) and 20.75% (B3) in control. The abundance of *Ruminococcaceae* was increased from 8.78% (A1) to 12.67% (A3) in FMT treatments, while decreased in control from 11.20% (B1) to 8.99% (B3), and *Enterobacteriaceae* almost eliminated after FMT-treated 12 weeks (0.61%) while still enriched in control (10.20%). Aspect of genus level, the dominant *Prevotella* abundance was decreased from 27.83% (A1) to 23.89% (A3) in FMT treatments and from 36.55% reduced to 20.67% in control, while *Bacteroides* increased from 19.96% (A1) to 22.97% (A3) in FMT treatments and from 9.88% (B1) to 25.62% (B3) in control. The toxin releaser *Escherichia−Shigella* could be induce inflammation and the abundance declined from 1.93% (A1) to 0.39% (A3) but increased in control from 3.76% (B1) to 9.53% (B3) (Figure 2).

Similarly, previous study also identified the majority bacterial distributed in *Firmicutes* and *Bacteroidetes* (66% and 25%) in FMT-treated IBS patients [30–33]. Francis et al. reported the anxiety was related to *Bacteroidetes* (*Bacteroides*) and *Proteobacteria*, and elevated abundance of *Proteobacteria* highly occurred in depression patient, while lower *Firmicutes*/*Bacteroidetes* ratio observed in psychological distress [23]. El-Salhy et al. observed the changes in bacterial abundance in FMT-treated IBS patients, increased in *Alistipes, Bacteroides, Prevotella, Firmicutes*, and *Akkermansia muciniphila* while decreased in *Eubacteriumhallii* and *Dorea* [19]. Canakis et al. proposed the resembled bacterial abundance in initially, while clearly altered in *Actinobacteria, Ruminococcusgnavus,* and *Bifidobacteria* after 3 weeks therapy, remarkably decreased in *Actinobacteria* and *Bifidobacteria* after 20 weeks FMT-treated IBS [34]. The positive effect of FMT was verified in present study by lower abundance of *Proteobacteria* (3.02%), *Actinobacteria* (0.35%), *Bacteroides* (22.97%), and higher *Firmicutes*/*Bacteroidetes* (0.90%), *Bacteroidetes* (50.60%), and *Prevotella* (23.89%) after FMT-treated 12 weeks of IBS-D patients. Therefore, the introduced healthy flora into the gut of participants restores the ecological imbalance by improved the beneficial *Bacteroidetes* and *Firmicutes* and suppressed the pathogenic or toxic releaser *Enterobacteriaceae, Bacteroides* and *Escherichia−Shigella*.

### 3.3. Alteration of alpha and beta diversity after fecal microbiota transplantation therapy IBS-D patients

The intestinal microbial community maintains dynamic balance to form a stable intestinal micro-ecosystem, which participates in the differentiation and proliferation of intestinal mucosal epithelial cells and promotes the development of the mucosal immune system [34]. While intestinal microbial disordered and relative abundance of bacterial dis-balance in proportions, such as reduced probiotics and increased pathogenic bacteria including *Escherichia coli* and *Bacillus*, which triggered various symptoms [35,36]. Several studies proposed that the increased alpha diversity and the proximity of beta diversity to the donor microbiota were predictors of successful FMT therapy [37,38].

**Figure 3.**
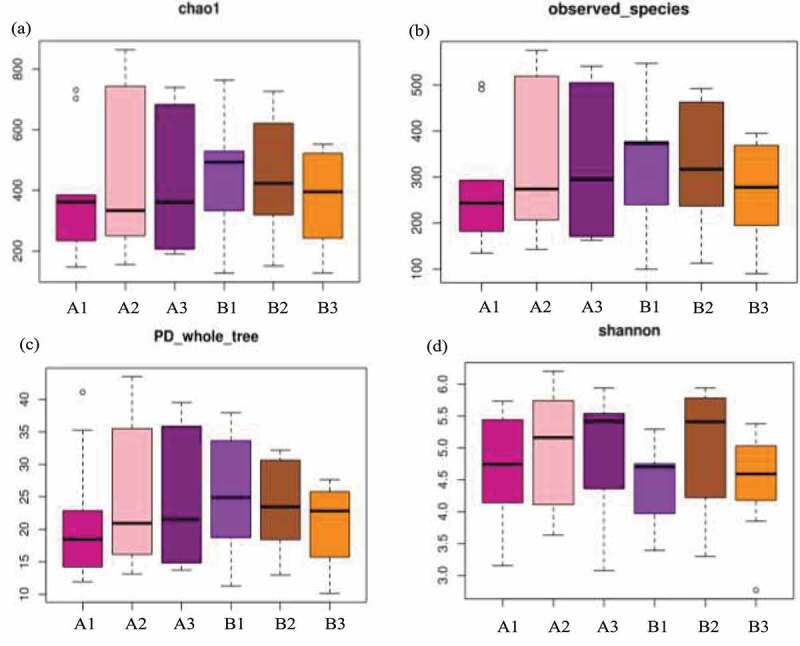
Alpha diversity of bacterial community-based chao1 (a), observed _species (b), PD whole tree (c) and Shannon (d). The FMT capsules treated IBS-D patients 1 week (A1), 8 weeks (A2), and 12 weeks (A3), as well as control with oral empty capsules 1 week (B1), 8 weeks (B2), and 12 weeks (B3)

**Figure 4.**
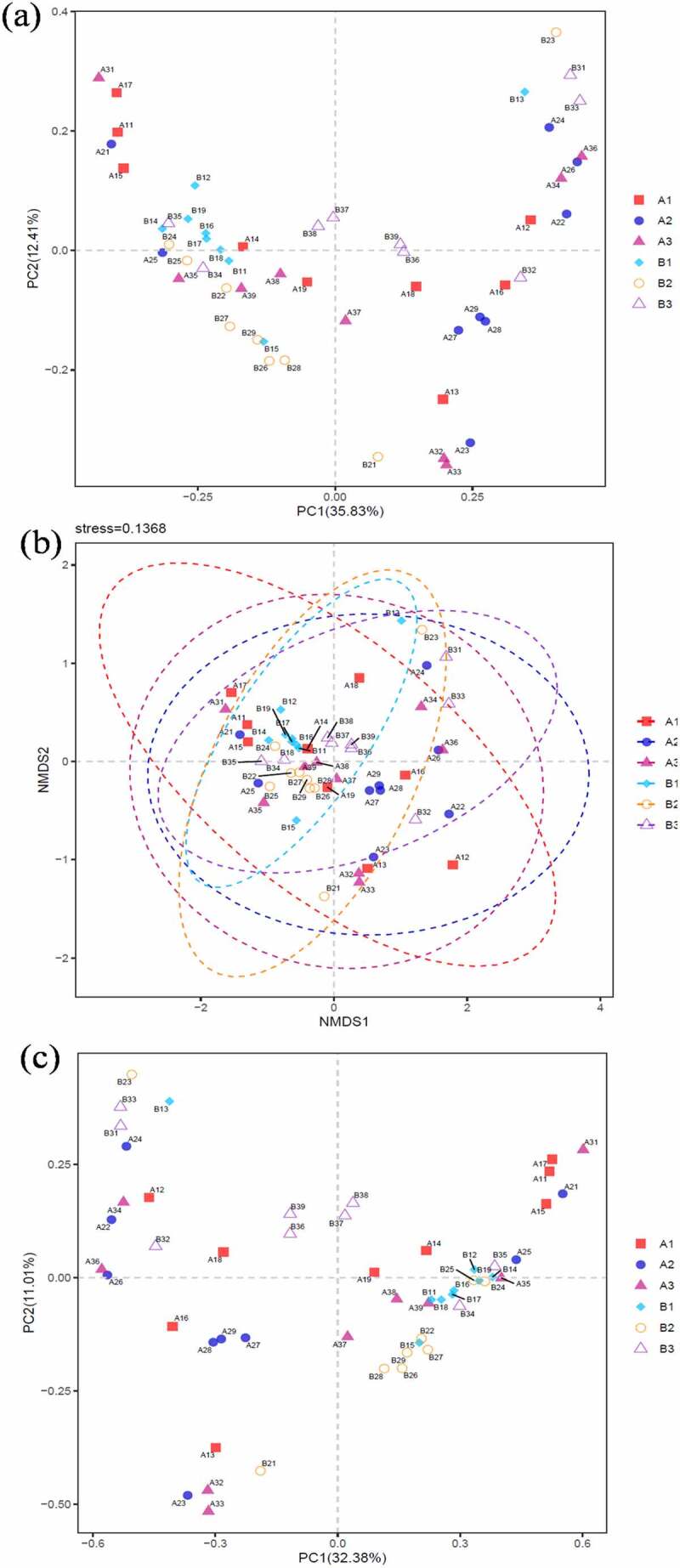
Beta diversity-based bacterial principal co-ordinates (a), nonmetric multidimensional scaling (b), and principal component analysis (c). The FMT capsule-treated IBS-D patients 1 week (A1), 8 weeks (A2), and 12 weeks (A3), as well as control with oral empty capsules 1 week (B1), 8 weeks (B2), and 12 weeks (B3)

**Figure 5.**
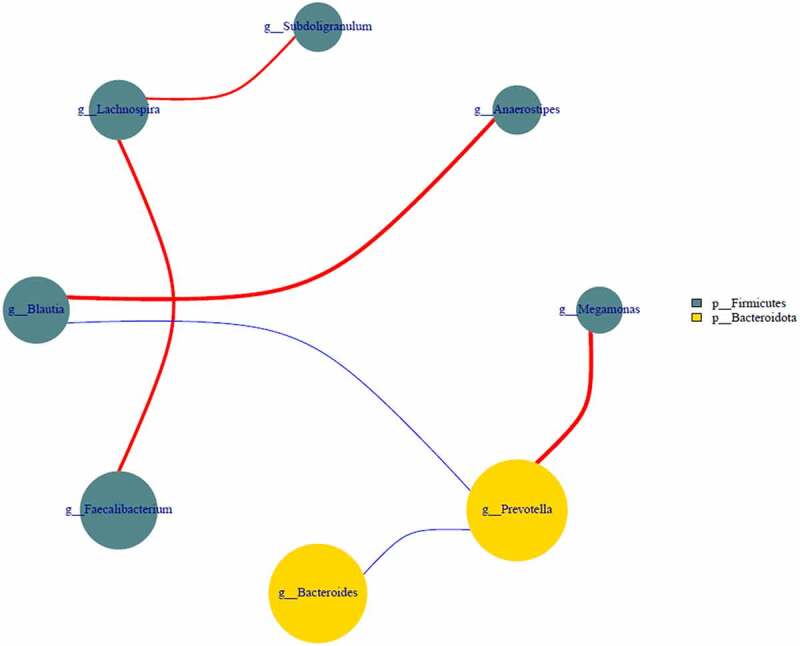
The correlation of selected bacterial community-based network analysis

Present study identified the positive effect of FMT therapy on bacterial diversity. Specifically, the number of OTUs actually observed was higher in FMT treatments and elevated from 277 (A1) to 329 (A2) and 331 (A3) compared with control from 332 (B1) declined to 316 (B2) and 282 (B3). The estimated number of OTUs of chao1 value was higher in FMT treatments from 380 (A1) to 447 (A2) and 424 (A3) than control of 452 (B1), 430 (B2) and 385 (B3). Additionally, the higher value of PD whole tree in FMT treatments of 22 (A1), 25 (A2) and 24 (A3) compared with control of 25 (B1), 23 (B2) and 21 (B3), as well as higher Shannon value of 4.67 (A1), 4.97 (A2) and 4.95 (A3) in FMT treatments than control of 4.12 (B1), 4.92 (B2) and 4.51 (B3) (Figure 3). On comparison, the alpha diversity index between FMT-treated group and control concluded that the FMT therapy elevated the bacterial diversity of the intestinal microbiota and enriched with therapy duration compare with control. In addition, as demonstrated in beta diversity, the distribution of intestinal bacterial community during FMT was dispersed obviously, closely cluster observed in FMT-treated 8 weeks and 12 weeks (A2 and A3), while the control microbial distribution was concentrated among FMT-treated 1, 8, and 12 weeks (Figure 4), that indicated the altered bacterial community contributed by FMT therapy and similar result also identified in previous studies [39,40]. Moreover, the correlation among dominant genus demonstrated that *Prevotella* was positively with *Megamonas* (0.62), negatively with *Bacteroides* (−0.83) and *Blautia* (−0.65). *Lachnospira* was positively with *Faecalibacterium* (0.55) and *Subdoligranulum* (0.74). *Blautia* was positively with *Anaerostipes* (0.62) and negatively with *Prevotella* (−0.54) (Figure 5). Simpson et al. pointed out the depression was negatively with *Lachnospiraceae* as well as positively with *Proteobacteria* and *Prevotellaceae*, and anxiety was positively related with *Anaerotruncus* [1]. El-Salhy et al. identified the IBS-SSS score was related with *Lactobacillus* (−0.3), *Alistipes* (−0.3), *Eubacterium biforme* (0.03), and *Bacteroides* (0.06) [19]. However, this study cannot identified the accurately bacterial species associated with clinical condition of IBS-D combined with anxiety and depression due to limited samples, and further study need to expand the sample range and clarify the correlation between specific bacteria and clinical indicators. Additionally, Johannes et al. reported that the high abundance of *Proteobacteria* was recorded in depressive disorder and contributed as a feature for IBS, and *Prevotellaceae* was related with IBS severity [24], while the reduced abundance in this study was supported the FMT positive therapeutic effect. Antushevich et al. proposed the positive effect of FMT on IBS by adjusted clinical symptoms and decreased relative abundance of *Proteobacteria* and increased in *Bacteroides* and *Firmicutes*, meanwhile fecal characteristics changes were related to increased *Firmicutes* and *Clostridia* and decreased *Bacteroidetes* [41–45]. Overall, FMT therapy increased bacterial diversity and adjust the bacterial community distribution, which further influence clinical and psychiatric symptoms, and FMT could be considered as potential and promising therapy for IBS-D combined with anxiety and depression to restore the intestinal micro-ecology.

## 4. Conclusion

The positive therapeutic effect of oral FMT alleviated the IBS-D severity and psychological depression/anxiety and altered bacterial community. The FMT therapy relieved the clinical symptoms of IBS-D patients by decreased the IBS-SSS score (113 points) and mitigated anxiety and depression score (9 and 4 points). Introduced healthy microbiota restores the gut ecological imbalance by enriched bacterial diversity, adjusted the community distribution, including improved the beneficial *Bacteroidetes* and *Firmicutes* and suppressed the toxic releaser *Enterobacteriaceae, Bacteroides* and *Escherichia−Shigella*. In short, FMT therapy has great potential for IBS-D patients combined with anxiety and depression to restore the intestinal micro-ecology and alleviate the clinical symptom.

## Highlights

- The bacterial diversity increased after fecal microbiota transplantation (FMT).
- Evaluated the response of diarrheal irritable bowel syndrome (IBS-D) to FMT.
- The FMT has positive therapeutic effect on IBS-D severity and depression/anxiety.
- The FTM therapy elevated the relative abundance of *Bacteroidetes* and *Firmicutes*.
- FMT therapy has great potential for IBS-D comorbid with anxiety and depression.

## List of abbreviation

Bowel syndrome quality of life Scale: IBS-QOL

Depression Scale: HADS

Fecal microbiota transplantation: FMT

Hamilton Anxiety Rating Scale: HAM-A

Hamilton Depression Rating Scale: HAM-D

Irritable bowel syndrome: IBS

Irritable bowel syndrome with predominant diarrhea: IBS-D

Irritable bowel syndrome scoring system: IBS-SSS

Operational Taxonomic Units: OTUs

Pair-End: PE
